# Visual Perceptual Learning Induces Long-Lasting Recovery of Visual Acuity, Visual Depth Perception Abilities and Binocular Matching in Adult Amblyopic Rats

**DOI:** 10.3389/fncel.2022.840708

**Published:** 2022-04-26

**Authors:** Alan Consorti, Gabriele Sansevero, Claudia Torelli, Irene Di Marco, Nicoletta Berardi, Alessandro Sale

**Affiliations:** ^1^Department of NEUROFARBA, University of Florence, Florence, Italy; ^2^Neuroscience Institute, National Research Council (CNR), Pisa, Italy

**Keywords:** visual cortex plasticity, amblyopia, perceptual learning, stereopsis impairment, active training

## Abstract

An abnormal visual experience early in life, caused by strabismus, unequal refractive power of the eyes, or eye occlusion, is a major cause of amblyopia (lazy eye), a highly diffused neurodevelopmental disorder severely affecting visual acuity and stereopsis abilities. Current treatments for amblyopia, based on a penalization of the fellow eye, are only effective when applied during the juvenile critical period of primary visual cortex plasticity, resulting mostly ineffective at older ages. Here, we developed a new paradigm of operant visual perceptual learning performed under conditions of conventional (binocular) vision in adult amblyopic rats. We report that visual perceptual learning induced a marked and long-lasting recovery of visual acuity, visual depth perception abilities and binocular matching of orientation preference, and we provide a link between the last two parameters.

## Introduction

Amblyopia is caused by an early and protracted unbalance in the activity of the two eyes due to refractive defects, cataract or strabismus, and is a major cause of impaired vision, with a prevalence of 1–5% (Holmes and Clarke, 2006). Treatment of the two fundamental deficits associated with amblyopia, i.e., impaired stereoscopic depth perception and reduced visual acuity (Webber and Wood, 2005; Levi et al., 2015), is very hard in adult subjects, due the remarkable decline in visual cortex plasticity at the end of the so-called critical period (CP) (Morishita and Hensch, 2008; Bavelier et al., 2010; Sale and Berardi, 2015).

New emerging strategies are starting to support the possibility to force the brakes that limit plasticity in the primary visual cortex (V1), favoring sufficient recovery of visual functions even in adult amblyopic subjects (Pizzorusso et al., 2006; Sale et al., 2007; Morishita and Hensch, 2008; Bavelier et al., 2010; Baroncelli et al., 2011; Montey and Quinlan, 2011; Hübener and Bonhoeffer, 2014; Kaneko and Stryker, 2014; Lunghi and Sale, 2015; Sale and Berardi, 2015; Lunghi et al., 2018).

Focusing on non-invasive strategies based on behavioral manipulations, visual perceptual learning (vPL), defined as the long-lasting change in visual discrimination abilities that results from visual practice, appears as particularly promising. In human amblyopic subjects, vPL can remarkably enhance visual functions on a wide range of tasks (Levi and Li, 2009; Astle et al., 2011), including Vernier acuity (Levi and Polat, 1996; Levi et al., 1997), positional acuity (Li and Levi, 2004; Li et al., 2005, 2007), contrast sensitivity (Polat et al., 2004; Zhou et al., 2006; Huang et al., 2008), and first-order letter or second-order letter identification (Levi, 2005; Chung et al., 2006, 2008). To date, there are only few studies concerning vPL in animal models. Thus, the available knowledge of the mechanisms underlying the beneficial effects of this treatment remains very limited. A form of experience-dependent response enhancement was described in the visual cortex of awake mice (Frenkel et al., 2006), in which repeated presentations of grating stimuli of a single orientation resulted in a persistent enhancement of responses evoked by the test stimulus. Being based on a passive viewing of visual stimuli, this procedure lacks the active component of vPL. Using a forced-choice active task based on swimming and discrimination of visual gratings in rats (Sale et al., 2011; Bonaccorsi et al., 2014), we demonstrated that vPL promotes synaptic potentiation of neuronal connections in V1 (Sale et al., 2011), and, when practiced with the amblyopic eye, favors recovery of visual functions in amblyopic rats past the end of the CP (Baroncelli et al., 2012).

Despite the recognized capability of vPL to enhance plasticity and favor recovery from amblyopia, several crucial issues remain to be addressed. Very little is known about the effects of vPL on visual depth perception abilities. This is partly due to the fact that vPL has been mostly tested, in animal models, with a reverse occlusion approach, in which the subjects are required to perform a visual task with the amblyopic eye open and with the concomitant occlusion of the fellow eye. In contrast to conventional occlusion therapies, binocular training approaches that require both eyes to cooperate are particularly promising in adult amblyopic patients (Hess et al., 2010, 2014; Li et al., 2013). Moreover, very little is known on the cellular mechanisms possibly involved in the effects elicited at the visual depth perception level, a fundamental issue when considering the relevance of stereopsis deficits in the visual impairment of amblyopic subjects. Finally, the long-term maintenance of visual function recovery after the end of perceptual training, an essential clinical requirement, has not been investigated so far.

Here, we focused on these still unsolved issues, exploiting our operant vPL paradigm in adult amblyopic rats with ordinary (binocular) viewing conditions.

## Materials and Methods

### Animal Treatment

The experiments were conducted on Long-Evans black hooded rats, in accordance with the approved guidelines and regulations of Italian Ministry of Public Health (approved protocol n. 16C). Animals were housed in a room with a temperature of 21°C and a 12-h light–dark cycle, with food and water available *ad libitum*.

### Surgery and Experimental Procedures

Rats were anesthetized with zolazepam + tiletamine (Zoletil, 1 mg/kg) and monocular deprivation (MD) was performed through eyelid suture at postnatal day (P) 21. Lid margins were trimmed and sutured with 6-0 silk. A post-operative health check control was performed daily until complete cicatrization; subjects with spontaneous re-opening were excluded. At P70, the long-term deprived eye was re-opened under anesthesia using thin scissors. After reopening of the deprived eye, rats were divided in two groups according to the experimental protocol: vPL and control animals. A third group of naïve, age-matched animals was added as control group. In all conditions, the animals were maintained in standard housing conditions, consisting of 40 × 25 × 20 cm cages (three animals per cage).

#### Visual Perceptual Learning Task

We used a modified version of the visual water box task (Prusky et al., 2000; Sale et al., 2011). Briefly, the animals were trained to distinguish a low spatial frequency grating (reference grating, 0.116 c/deg) from a higher spatial frequency grating (test grating, 0.592 c/deg). When the animals achieved a level of >80% of accuracy in at least three subsequent sessions (criterion level), the vPL protocol was started by gradually reducing the spatial frequency of the test grating. Thus, the test grating became progressively more similar to the reference grating. For each animal, a daily threshold was calculated as the average spatial frequency of the test grating that the rat was able to distinguish (at least 70% correct performance) from the reference grating within the different sessions. The vPL task ended when the animal performance reached a plateau (performance at a given spatial frequency of the test grating oscillating around 70% of correct choices for three consecutive days). A group of control animals (CTRL) was trained to distinguish the reference grating from a test grating whose spatial frequency was always maintained at the starting value of 0.592 c/deg (associative test). CTRL rats were matched to vPL animals in terms of overall swim time and training days in the water maze.

#### Behavioral Assessment of Visual Functions

##### Visual Acuity

We used *n* = 7 vPL and *n* = 8 CTRL rats. We first measured visual acuity of the fellow eye (not deprived). Then, we measured visual acuity of the amblyopic eye (by temporary occlusion of the fellow eye) five times, i.e., immediately after eyelid reopening (at P70), immediately at the end of the perceptual learning or control task, and then at 30, 90, and 180 days past the end of the procedure. To measure visual acuity, we used the visual water task (Prusky et al., 2000; Sale et al., 2007), which trains animals to first distinguish a low (0.1 c/deg) spatial frequency vertical grating from grey, and then tests the limit of this ability at higher spatial frequencies. Once 80% accuracy was achieved, the limit of the discrimination was estimated by increasing the spatial frequency of the grating. Animals were trained 60 trials per day until the achievement of discrimination limit criteria (Prusky et al., 2000). Visual acuity was calculated as the spatial frequency corresponding to 70% of correct choices on the sigmoidal function fitting the psychometric function. During each session, the experimenter was blind to the experimental group.

##### Visual Depth Perception

We used *n* = 8 vPL and *n* = 6 CTRL rats. Visual depth perception was assessed as spontaneous exploration in the visual cliff apparatus, as previously described (Baroncelli et al., 2013; Sansevero et al., 2019). Briefly, the arena was divided into a shallow and a deep side. On the shallow side, a patterned floor was positioned immediately below the glass plate, while on the deep side the checked platform was moved to 29 cm below the glass plate. Each animal was placed on the shallow side, and the total time the rat spent exploring each side of the arena was automatically recorded by the Noldus EthoVision system. The trial ended after 5 min. The arena was cleaned between trials with an alcohol solution. An exploration index was calculated as follows: (ts-td)/ttot, where ts and td are, respectively, the time spent exploring the shallow side and the deep side of the arena, and ttot is the total time of the test procedure. Each animal was tested only once.

##### *In vivo* Electrophysiology

Electrophysiological recordings were performed as previously described (Baroncelli et al., 2016; Mazziotti et al., 2017; Sansevero et al., 2020). Rats were anesthetized with i.p. injection of urethane (1.4 g kg^–1^, i.p., 20% in saline; Sigma-Aldrich) and placed on a stereotaxic frame, with the body temperature maintained at 37°C. A craniotomy was performed over the binocular visual cortex (4.8–5.2 mm lateral to lambda) and the dura mater was removed. The two eyes were fixed and kept open by means of adjustable metal rings surrounding the external portion of the eye bulb. An electrode (2 × 2-tet-3 mm-150–150-121-A16-15, Neuronexus Technologies) was lowered into the cortex to record local field potentials and single-unit activity. Signals were acquired using a 16 channels Neuralynx device and data analysis was performed using a custom MATLAB software. Visual stimuli were generated in MATLAB using Psychophysics Toolbox extension and displayed, with gamma correction, on a monitor (Sony Trinitron G500, 60 Hz refresh rate, 32 cd m^–2^ mean luminance) placed 20 cm in front of the animal.

For visual evoked potentials (VEPs), extracellular signal was filtered from 0.1 to 275 Hz and sampled at 20 kHz. VEPs in response to sinusoidal wave patterns with a spatial frequency of 0.03 c/deg and abrupt phase inversion (0.5 Hz temporal period), were evaluated in the time domain by measuring the peak-to-baseline amplitude and latency. VEPs were acquired using the responses coming only from the tetrode in the up-right position inside the inserted probe (responses from the four contact points were averaged together), at 100 μm of depth first, and then at 400 μm, corresponding to layers 2, 3, and 4, respectively. Computer controlled mechanical shutters were used to alternatively close the two eyes. Visual acuity was obtained by extrapolation to zero amplitude of the linear regression through the data points in a curve where VEP amplitude was plotted against log spatial frequency. We measured ocular dominance by calculating the contralateral to ipsilateral VEP ratio (C/I ratio), i.e., the ratio of VEP amplitudes recorded by stimulating the eye contralateral and ipsilateral, respectively, to the visual cortex where the recording is performed. Recordings were performed only from the visual cortex contralateral to the previously deprived eye (amblyopic eye). During recording through one eye, the other was occluded by an automatic black shutter. The C/I VEP ratios obtained at the two depths did not differ between each other, and have been averaged together.

For single-unit recordings, extracellular signal was filtered from 0.6 to 9 kHz and sampled at 30.3 kHz. Only cells with receptive fields within 20° from the vertical meridian were included in our sample. Spiking events were detected online by voltage threshold crossing and waveforms of 1 ms were acquired around the time of threshold crossing. To improve single unit isolation, recordings from groups of four neighboring sites (tetrode) were linked, so that each spike was composed by four waveforms. Data were loaded on the Offline Sorter software (Plexon), and a principal component analysis was performed to score spikes with a high degree of similarity in a 3D feature space. Waveforms from each electrode of the tetrodes were processed together to improve isolation. Clusters were progressively defined using convex hulls and then recalculating principal component analysis. Quality of separation was determined based on the following criteria: (1) during manual clusterization with convex hulls, raw waveforms in the clusters were visually inspected to check the uniformity of single waveforms; (2) clusters contained < 0.1% of spikes within a 1.0 ms interspike interval; (3) auto- and cross-correlograms of the clusters were also inspected to reveal if the cluster contained more than a single unit or if several clusters contained spikes of the same unit; and (4) the peak amplitude of a unit remained stable over the entire recording session. Units were included in the sample for analysis of tuning properties when they had an average peak firing rate, across trials of the optimal stimulus for the dominant eye, of > 0.5 Hz. Drifting sinusoidal gratings were used as visual stimuli (1.5 s duration, temporal frequency of 2 Hz, 12 different orientations with a step of 30°, 3 spatial frequencies: 0.02, 0.04, 0.08 c/deg). Stimulation was repeated five times per eye, with stimulus conditions randomly interleaved, and two grey blank conditions (mean luminance) were included in all stimulus sets to estimate the spontaneous firing rate. The average spontaneous rate for each unit was calculated by averaging the rate over all blank condition presentations. Responses at each orientation and spatial frequency were calculated by averaging the spike rate during the 1.5 s presentation and subtracting the spontaneous rate. The preferred stimulus was determined finding the combination of spatial frequency and orientation that maximize the response, independently for each eye. Orientation tuning curves were constructed for the spatial frequency that gave maximal response at this orientation. Given this fixed preferred orientation (OPref), the tuning curve was fitted as the sum of two Gaussians centred on OPref and OPref + π, of different amplitudes but equal width, with a constant baseline. From this fit, we calculated an orientation selectivity index (OSI) representing the ratio of the tuned versus untuned components of the response, and the width of the tuned component. OSI was calculated as follows: (respOPref-respOOrtho)/(respOPref + respOOrtho), where resp is the maximal response evoked by visual stimulation and OOrtho is the orientation orthogonal to the preferred one. Tuning width is the half-width at half-maximum of the principal gaussian. In addition, we also calculated a direction selectivity index (DSI), as follows: (respOPref-respOOppo)/(respPref + respOppo). The difference in preferred orientation between the two eyes (binocular matching, ΔO) was calculated by subtracting ipsilateral OPref from contralateral OPref along the 180°cycle.

### Statistical Analyses

Statistical analysis was done using SigmaStat Software. Data were tested for normality before running statistical tests; parametric tests were run on normally distributed data and, in case normality test failed, non-parametric tests were performed as appropriate. Differences between two independent groups were assessed with a two-tailed *t*-test; differences between two dependent groups (e.g., visual acuity of the deprived and fellow eyes in the same animals) were assessed with a two-tailed paired *t*-test. One-way ANOVA, One-way RM ANOVA, and Two-way RM ANOVA, followed by Holm-Sidak multiple comparison procedure, were used to compare normally distributed data belonging to more groups. One-way ANOVA on ranks, followed by Dunn’s method or Tukey test, were performed to compare not normally distributed data belonging to more than two groups. Level of significance was *p* < 0.05, unless otherwise specified. The size of biological replicates is indicated by the n numbers in the various experimental sections.

## Results

**FIGURE 1:**
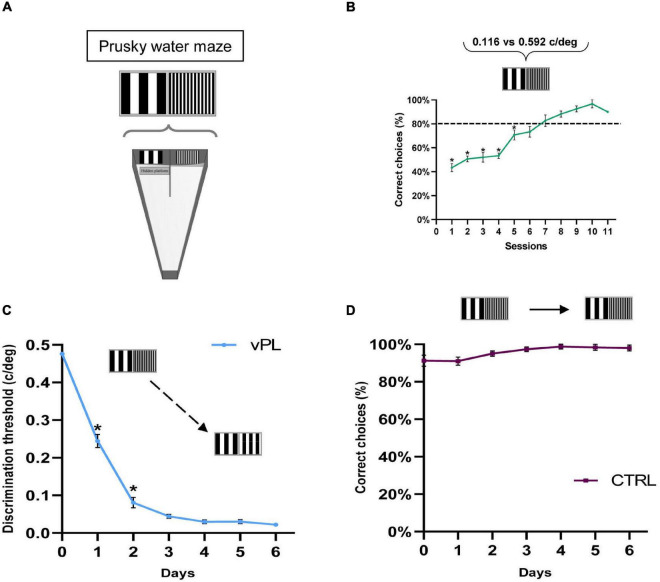
Visual perceptual learning in adult amblyopic rats. **(A)** A modified version of the visual water box task was used to perform vPL. **(B)** Mean performance in distinguishing a test grating of 0.592 c/deg from the standard grating (0.116 c/deg) across the training sessions. All animals (both vPL and CTRL rats) have been pulled together. The increase in the percentage of correct choices with sessions is significant (One-way RM ANOVA on ranks *p* < 0.001). **(C)** Improvement of discrimination threshold in vPL rats involved in the visual discrimination task. The threshold, calculated as the minimum spatial frequency difference between the reference and the test gratings discriminated, decreases significantly with the training days (One-way RM ANOVA on ranks *p* < 0.001). **(D)** The performance of CTRL animals involved in a discrimination task lacking the incremental component remained stable across the training days. Error bars indicate s.e.m.; * indicates statistical significance.

We investigated the effects of vPL on visual function recovery in adult amblyopic rats, by assessing visual abilities in adult animals subjected to long-term monocular visual deprivation started at the peak of the CP for plasticity (Postnatal day 21, P21), and then exposed, after reopening of the previously deprived eye at 2 months of age, to a forced-choice vPL task performed in binocular sight conditions (vPL rats). The task required subjects to distinguish between two vertical gratings differing only for their spatial frequency (Figures 1A,B), with a fixed low spatial frequency reference grating (0.116 c/deg), and a higher spatial frequency test grating (0.592 c/deg). When the animals achieved a level of >80% of accuracy in at least three subsequent sessions (criterion level), the vPL protocol was started by gradually reducing the spatial frequency of the test grating, providing an incremental training for the discrimination abilities of the animals. The performance of vPL rats was compared with that of control (CTRL) rats exposed to a simplified version of the same task, in which the incremental training phase was lacking. vPL rats displayed robust visual PL, as evidenced by the progressive reduction in the minimum discriminable spatial frequency difference (MDSFD) between the two gratings across the learning days (Figures 1C,D) (One-way RM ANOVA on ranks *p* < 0.001).

**FIGURE 2:**
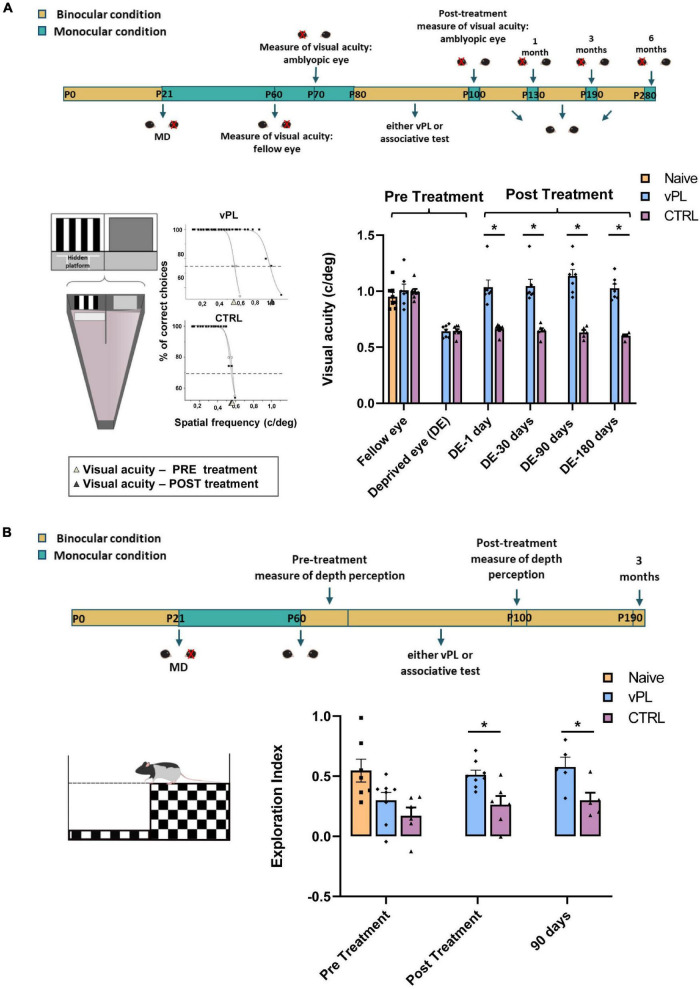
Visual perceptual learning induces enduring recovery of visual functions. **(A)** Top panel: schematic diagram of the protocol. Left panel: visual acuity through the long-term deprived and the fellow eye was measured using the visual water box task; example of sigmoidal extrapolations of psychometric curves used to calculate visual acuity are also reported. Right panel: visual acuity of the previously deprived eye was significantly different from that of the fellow eye in CTRL rats (Two-way RM ANOVA, *p* < 0.001), but not in vPL animals (*p* = 0.297). Two-way RM ANOVA on ranks revealed that, in vPL rats, visual acuity of the previously deprived eye immediately after the end of the perceptual learning task was significantly increased with respect to that measured before treatment (*p* < 0.001), and remained unaltered 30, 90, and 180 days after the end of the treatment (*p* = 0.817, *p* = 0.095 and *p* = 0.958, respectively). In contrast, the visual acuity of the long-term deprived eye did not change throughout the study in CTRL rats (Two-way RM ANOVA on ranks with Holm-Sidak method, pre-treatment vs. post-treatment, *p* = 0.999; pre-treatment vs. 1 month after treatment, *p* = 0.982; pre-treatment vs. 3 months, *p* = 0.964 and pre-treatment vs. 6 months, *p* = 0.997). **(B)** Top panel: schematic diagram of the protocol. Left panel: visual depth perception was assessed using the visual cliff task, as the exploration preference for the shallow and depth side of the arena. Right panel: one-way ANOVA showed a significant preference for the shallow side in vPL animals, which exhibited an exploration index statistically higher than that of CTRL rats at all time-points (Holm-Sidak method, *p* < 0.05). All animals were tested after restoration of binocular vision. Error bars indicate s.e.m.; * indicates statistical significance.

To assess the impact of vPL on visual function recovery, we first measured visual acuity behaviorally (the experimental protocol is depicted in Figure 2A), using the visual water-box task (Prusky et al., 2000; Sale et al., 2007), in which the rats are first conditioned to distinguish a low spatial frequency grating from a homogeneous gray, with high reliability (training phase), and then tested for their capability to discriminate higher spatial frequencies (Figure 2A). Using this task longitudinally in the same individuals, we measured visual acuity through the previously deprived eye five times, i.e., immediately before treatment, immediately at the end of vPL, and at three additional follow-ups (1, 3, and 6 months past the end of the treatment). While a complete visual acuity recovery was evident in vPL animals (*n* = 7) (amblyopic eye: 1.03 ± 0.06 c/deg; fellow eye: 1.00 ± 0.05 c/deg), no recovery was instead found in CTRL rats (*n* = 8; amblyopic eye: 0.66 ± 0.01 c/deg; fellow eye: 0.99 ± 0.02 c/deg) (Two-way RM ANOVA, treatment × time *F* = 78,526, DF = 1, Holm-Sidak method, *p* = 0. 297 for vPL rats, *p* < 0.001 for CTR rats) (Figure 2A). Strikingly, visual acuity recovery in vPL rats was long-lasting, as it was maintained at all time points beyond the end of the vPL procedure (Two-way RM ANOVA on ranks, Holm-Sidak method, post-treatment vs. 1 month after treatment, *p* = 0.817; post-treatment vs. 3 months, *p* = 0.095; post-treatment vs. 6 months, *p* = 0.958) (Figure 2A). At the end of the study, visual acuity measured in the formerly amblyopic eye of vPL animals was not significantly different from that of an additional group of non-deprived and untreated rats (naïve animals), while both were significantly higher than visual acuity values measured in CTRL rats (naïve, 0.95 ± 0.03 c/deg; One-way ANOVA, *F* = 32.783, DF = 2, Holm-Sidak method, *p* = 0.143, *p* < 0.001, *p* < 0.001, respectively).

Then, we focused on visual depth perception abilities, using the visual cliff task, which exploits the spontaneous tendency of rodents to avoid the deep side of a visual cliff (the experimental protocol is depicted in Figure 2B). Discrimination between the deep and the shallow side of the arena requires an intact binocular vision, as rats with either acute monocular occlusion or with amblyopia are known to display no preference for the shallow side, while rats with binocular vision do (Baroncelli et al., 2013). Accordingly, CTRL rats were impaired in their visual depth perception, and showed no preference for the shallow side of the arena (*n* = 6; exploration index = 0.26 ± 0.07); in contrast, vPL rats (*n* = 8) displayed a clear preference for the shallow side, with an exploration index (0.51 ± 0.03), significantly higher than that of CTRL rats (Figure 2B), but not significantly different from that of naïve rats (*n* = 7; 0.54 ± 0.09) (One-way ANOVA, between groups *F* = 4.329, DF = 2, Holm-Sidak method, *p* = 0.041, *p* = 0.046, *p* = 0.728, respectively). In agreement with the results of visual acuity, recovery of visual depth perception abilities in vPL rats turned out to be long-lasting, persisting for the entire 3-month period of follow-up analysis (Figure 2B). (Two-way repeated measure ANOVA, *F* = 1.035, DF = 2, Holm-Sidak method, time among vPL, pre-treatment vs. post-treatment, *p* = 0.021; pre-treatment vs. 3 months, *p* = 0.025; post-treatment vs. 3 months, *p* = 0.801).

**FIGURE 3:**
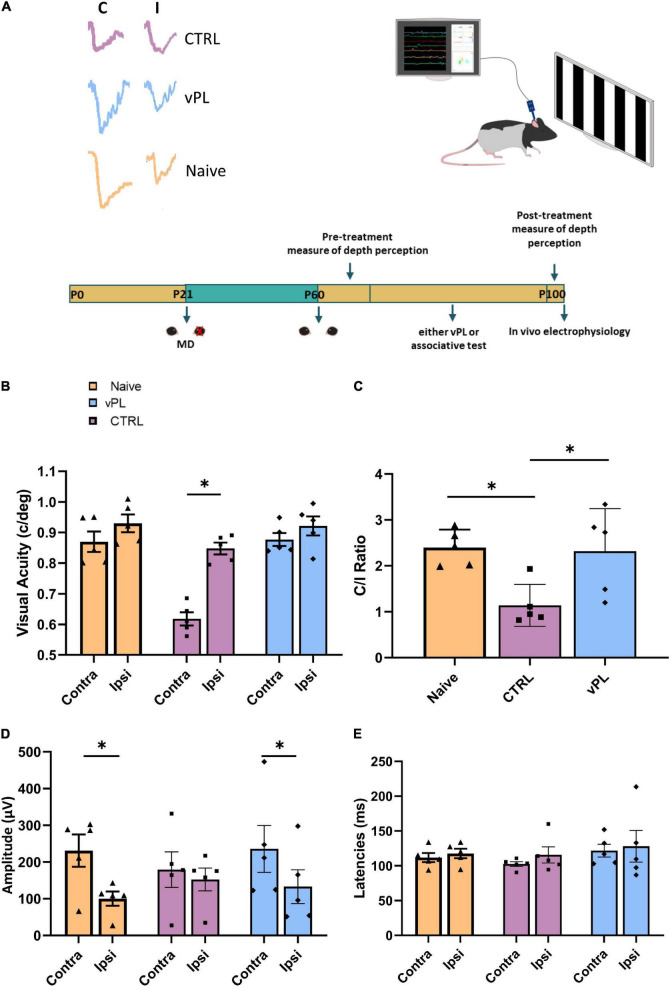
**(A)** Schematic diagram of the protocol. **(B)** Electrophysiological recordings of visual evoked potentials form the primary visual cortex. In CTRL rats, visual acuity of the deprived eye remained significantly lower with respect to the other eye (One-way RM ANOVA, Holm-Sidak method, *p* < 0.001); in contrast, a full visual acuity recovery was achieved by vPL rats (*p* < 0.001) with values not different from those of naïve animals (*p* = 0.433). **(C)** Ocular dominance was assessed through the C/I VEP ratio in response to a low spatial frequency grating. The C/I VEP ratio was significantly higher in vPL than in CTRL rats (One-way ANOVA, Holm-Sidak method, *p* = 0.025), but not different from that of naïve rats (*p* = 0.847). Error bars indicate s.e.m.; * indicates statistical significance. **(D)** Accordingly with the C/I ratio, the average amplitude of VEPs was higher for the contralateral eye with respect of the ipsilateral one, in both Naïve and vPL rats (Two-way RM ANOVA, *p* < 0.01 and *p* = 0.004, respectively); no difference was instead observed between the potentials of controlateral and ipsilateral eye in CTRL animals (*p* = 0.369). **(E)** No difference was observed for VEPs latencies across the different experimental groups (Two-way RM ANOVA, *p* = 0.560 for the factor treatment and *p* = 0.230 for the factor eye, without any interaction, *p* = 0.881). Error bars indicate s.e.m.; * indicates statistical significance.

Then, we used *in vivo* electrophysiology to monitor V1 visual evoked potentials (VEPs) in separate groups of animals, immediately after the end of the vPL procedure (the experimental protocol is depicted in Figure 3A). In CTRL rats (*n* = 5), visual acuity of the deprived eye remained significantly lower (0.63 ± 0.02 c/deg) with respect to the fellow eye (0.91 ± 0.03 c/deg) (Two-way RM ANOVA, Holm-Sidak method, *F* = 23.468, DF = 2, *p* < 0.001) (Figure 3B). In contrast, we found no difference between visual acuity values of the previously deprived vs. the fellow eye in both vPL rats (*n* = 5, contralateral visual acuity: 0.84 ± 0.02 c/deg; ipsilateral visual acuity: 0.84 ± 0.03 c/deg; *p* = 0.370 see Figure 3B), and in naïve animals (*n* = 5, contralateral visual acuity: 0.88 ± 0.02 c/deg; ipsilateral visual acuity: 0.94 ± 0.03 c/deg; *p* = 0.118). Moreover, visual acuity in the formerly amblyopic eye of vPL rats did not significantly differ from that of naïve animals, while it was significantly higher than that of CTRL rats (Figure 3B) (*p* = 0.433 and *p* < 0.001, respectively). Using VEPs, we also determined ocular dominance, by computing the so-called contralateral to ipsilateral (C/I) VEP ratio in response to a low spatial frequency grating (0.1 c/deg). In CTRL rats (*n* = 5), the C/I VEP ratio remained significantly lower than in naïve rats (*n* = 5) C/I VEP ratio in CTRL rats = 1.14 ± 0.2, in naïve rats 2.4 ± 0.17, (One-way ANOVA, Holm-Sidak method, *p* = 0.026), indicating the occurrence of a severe OD deficit (Figure 3C). In contrast, vPL rats (*n* = 5) had a C/I VEP ratio of 2.32 ± 0.41, significantly higher than that of CTRL rats (One-way ANOVA, Holm-Sidak method, *p* = 0.025), but not significantly different from that of naïve rats (*n* = 5; C/I VEP ratio = 2.4 ± 0.17; *p* = 0.847). Accordingly, VEPs average amplitude was higher for the contralateral eye with respect of the ipsilateral one in both Naïve and vPL rats (Figures 3D,E) (Two-way RM ANOVA, Holm-Sidak method, *F* = 27.261, DF = 2; Naïve: contra = 231.22 ± 44.11 μV, ipsi = 100.38 ± 19.32 μV; vPL: contra = 235.89 ± 64.1 μV, ipsi = 132.96 ± 45.97 ± V; *p* < 0.01 and *p* = 0.004, respectively); no difference was instead observed between the potentials of controlateral and ipsilateral eye in CTRL animals (contra = 179.43 ± 48.33 μV, ipsi = 152.49 ± 30.99 μV, *p* = 0.369). No difference was instead observed for VEPs latencies across the different experimental groups (Two-way RM ANOVA, *F* = 0.609, DF = 2, *p* = 0.560 for the factor treatment and *p* = 0.230 for the factor eye, without any interaction, *p* = 0.881).

**FIGURE 4:**
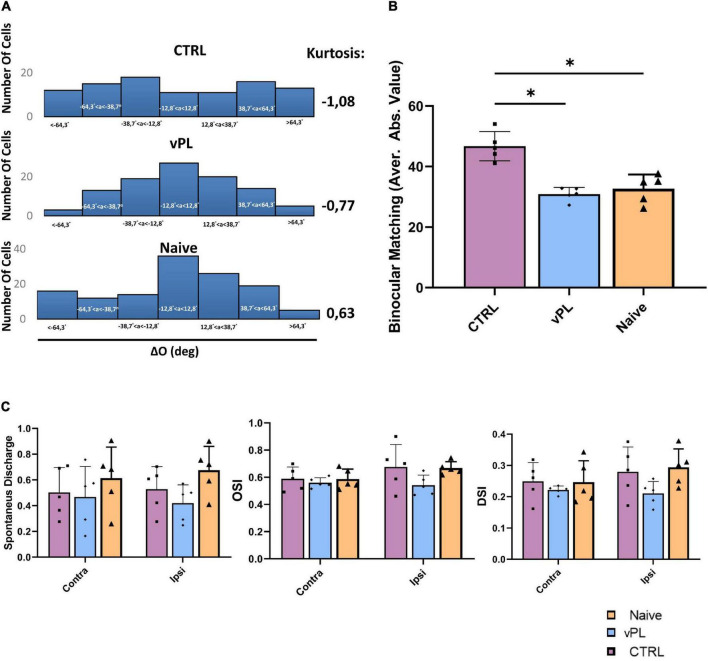
**(A)** Distributions of ΔO within the three experimental groups. **(B)** Comparison between average | ΔO| for the different groups; CRTL rats displayed higher | ΔO| in comparison with both vPL and Naïve animals (One-way ANOVA, Holm-Sidak method, *p* < 0.001 and *p* < 0.001, in both cases). Interestingly, no difference was observed between naïve and vPL rats (*p* = 0.498). **(C)** Assessment of spontaneous discharge, direction and orientation selectivity in V1. No difference was found in either the spontaneous discharge, the orientation selectivity index (OSI), or the direction selectivity index DSI among the three groups of animals (Two-way RM ANOVA, Holm-Sidak method, DF = 2, *F* = 1.075, *p* = 0.357; *F* = 0.682, *p* = 0.515; *F* = 0.199, *p* = 0.821, respectively). Error bars indicate s.e.m.; * indicates statistical significance.

Sensory deprivation started during the CP may permanently compromise the matching of individual cortical neurons’ orientation preferences through the two eyes, a fundamental step in the development of binocular vision (see Levine et al., 2017). Therefore, we used multichannel electrophysiological recordings (Mazziotti et al., 2017) of visual cortical single units to investigate whether the binocular properties of visual cortical neurons were affected in our amblyopic animals (CTRL rats), and whether the recovery of visual depth perception abilities induced by vPL was accompanied by a rescue of these binocular properties. Single units were recorded in anesthetized animals in response to drifting sinusoidal gratings that varied in orientation and spatial frequency, and we compared the distributions of single unit binocular matching of orientation preference (ΔO) in either vPL or CTRL rats, using the same animals previously subjected to the behavioral visual cliff analysis. In naïve animals, the majority of cells displayed a ΔO around 0 deg (in Figure 4A, central bin is −12.8° < ΔO < 12.8°; kurtosis = 0.63); while CTRL rats displayed a decreased kurtosis (kurtosis = −1.08) with respect to naïve animals, kurtosis levels were higher in vPL rats with respect to CTRL animals (kurtosis = −0.77) (Figure 4A), with a ΔO distribution very similar to that of naïve animals. Moreover, we compared the averages of ΔO absolute values (| ΔO|) among the three experimental groups (Figure 4B), and we found that CTRL rats displayed a higher | ΔO| (46.70° ± 2.16°) than both naïve (32.68° ± 2.1°) and vPL animals (30.77° ± 1°) (One-way ANOVA, Holm-Sidak method, *F* = 22.298, *p* < 0.001 and *p* < 0.001, respectively). Interestingly, no difference was observed between Naïve and vPL rats (*p* = 0.498). Equal variance of | ΔO| did not change across the three groups, either when computed on the average values of the subjects (*F*-test, *p* = 0.422), or when computed for all cells in the three different groups (Levene test, *p* = 0.115). To rule out the possibility that these effects at the level of ΔO were due to any change in terms of monocular selectivity to distinct features of the visual stimulus (orientation, direction), or to changes in terms of spontaneous activity, we measured the orientation selectivity index (OSI), the direction selectivity index (DSI) and levels of spontaneous activity in the two eyes, in all three experimental groups. No difference was found among the three groups of animals in either OSI, DSI or spontaneous activity (Figure 4C) (Two-way RM ANOVA, Holm-Sidak method, DF = 2, *F* = 1.075, *p* = 0.357; *F* = 0.682, *p* = 0.515; *F* = 0.199, *p* = 0.821, respectively).

**FIGURE 5:**
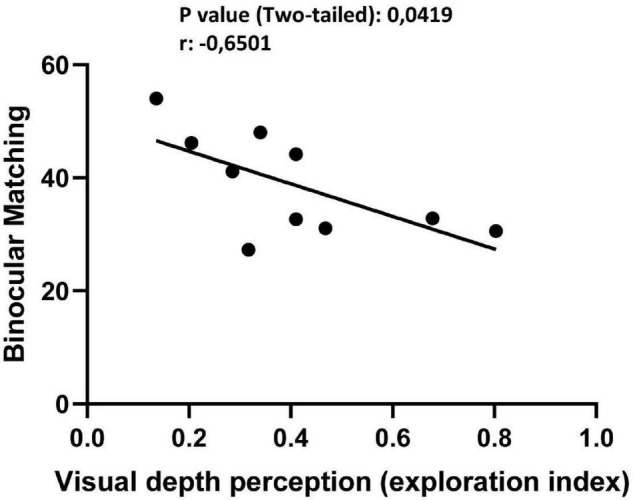
Significative correlation between | ΔO| and visual cliff scores suggests a causal link between binocular matching of orientation preference and depth perception abilities (Pearson *r* = –0.651, 95% confidence intervals –0.9080 to –0.03466, R squared = 0.4226, *p* = 0.0419).

Having shown both a behavioral effect on visual depth perception abilities using the visual cliff task and a rescue of ΔO distribution in single unit recordings from V1, we measured the relationship between these changes. For each vPL or CTRL subject used to record binocular matching, the mean |ΔO| between the two eyes for all single units analyzed at the preferred grating orientation was correlated with the exploration index in the visual cliff past the end of the behavioral procedure. We found that mean |ΔO | values correlated significantly with the visual cliff exploration index (Figure 5; Pearson *r* = −0.651, 95% confidence intervals −0.9080 to −0.03466, R squared = 0.4226, *p* = 0.0419). This correlation indicates that the better the binocular matching of orientation preference, the greater the visual depth perception abilities, suggesting a causal link between the two measures.

These results together show that vPL performed under conditions of unrestricted binocular vision induce a marked and enduring recovery of visual acuity, visual depth perception abilities and binocular matching in adult amblyopic rats.

## Discussion

We report that adult amblyopic rats subjected to vPL show a marked recovery of visual acuity, spanning a long follow-up period of stability. Indeed, the visual acuity recovery effect induced by vPL persisted for at least 6 months, quite a long time period when related to the rat life span. No recovery was instead found in control rats that only learned to discriminate a reference grating from a test grating that was always maintained at its initial spatial frequency value (CTRL group), suggesting that the component of incremental training is essential for the capability of visual learning to promote plasticity and visual function recovery.

Previously, a similar effect has been only reported in rats subjected to the vPL task under a reverse-occlusion condition (Baroncelli et al., 2012), a protocol that maximize the use of the amblyopic eye but is certainly limited in terms of application to human patients, due to potentially relevant risks (Hess and Thompson, 2015; Levi et al., 2015). There is an increasing consensus on the need to understand the cellular bases of sensory improvements documented from novel dichoptic therapies targeting binocular functions, such as videogames (Hess et al., 2010, 2011; Li et al., 2013) or passive dichoptic video viewing (Li et al., 2015a,b). In this context, the results reported in the present paper indicate a strong beneficial effect of vPL when performed without eye occlusion procedures, and encourage further studies based on binocular stimulation in adult amblyopic subjects. As a note of caution, however, it should be noted that the monocular deprivation approach used in our study simulates only deprivation human amblyopia, but not strabismic or refractive amblyopia, for which further studies in animal models are needed to assess the impact of vPL.

Importantly, the results obtained at the behavioral levels were fully confirmed with our electrophysiological analysis of VEPs. VEPs are considered the gold standard for visual acuity assessment, as their provide very accurate estimates of spatial discrimination values, in very good agreement with those obtained at the psychophysical level, in all species tested so far (e.g., Campbell and Maffei, 1970; Campbell et al., 1973; Ridder and Nusinowitz, 2006; Hamilton et al., 2021). Moreover, VEPs recordings allowed us to also measure ocular dominance by computing the so-called C/I VEP ratio, a fundamental property strongly affected in amblyopic subjects (Frenkel and Bear, 2004). Even if our results have been obtained in anesthetized animals, it has been previously reported that uretane anesthesia does actually only affect V1 processing of time-varying stimuli, leaving the spatial aspects of V1 receptive fields unchanged with respect to those of awake animals (see Durand et al., 2016).

While vPL had a remarkable impact on visual acuity, we did not find any effect on monocular OSI or DSI. This may be dependent on the difference between visual acuity and orientation/direction selectivity in terms of their dependence on visual experience during development. While selectivity of the primary visual cortex for spatial frequencies strongly depends on postnatal visual experience (see, for instance, Prusky et al., 2000; Prusky and Douglas, 2004), the orientation/direction preference is established just after eye-opening, independently of visual experience [see, for instance, (Wiesel and Hubel, 1974; Buisseret and Imbert, 1976; Wiesel, 1982; Fregnac and Imbert, 1984; Gödecke and Bonhoeffer, 1996; Thompson et al., 2008)]. Thus, long-term monocular deprivation started at the peak of the critical period was likely to strongly affect visual acuity, without exerting any effect on monocular OSI and DSI. Therefore, exposure of amblyopic animals to vPL might have improved visual acuity, leaving unaltered OSI and DSI.

Strikingly, we also found that vPL induced a remarkable recovery of visual depth perception abilities in the visual cliff task. This increases the potential translational interest of the results, as deficits in stereopsis constitute a very relevant impediment for normal everyday life activities in amblyopic subjects (Webber and Wood, 2005; Levi et al., 2015). The improvement in the visual cliff test induced by vPL was unlikely to be dependent on an improvement of monocular functions, like visual acuity. It has been previously shown, indeed, that naïve adult rats with one eye occluded but with normal vision are severely impaired in performing the same visual cliff task used in our manuscript (Baroncelli et al., 2013). Thus, monocular cues are not sufficient for a proper performance in the visual cliff task, even when the used eye is completely normal. Moreover, we did not find any difference in terms of monocular selectivity for direction or orientation among the three groups of animals. Lastly, the visual stimuli presented in the visual cliff were of very low spatial frequency, thus being fully perceivable also by amblyopic animals.

We also provide evidence that long-term monocular deprivation was associated with altered binocular matching properties for V1 cells, and that vPL, which is able to favor recovery of visual depth perception, was also accompanied by normalization of binocular matching in the primary visual cortex of amblyopic animals. Exposure to environmentally enriched conditions was previously shown to rescue binocular matching deficits caused by early visual deprivation in mice (Levine et al., 2017). Moreover, environmental enrichment does also rescue depth perception in adult amblyopic animals (Baroncelli et al., 2013). Here, we provide first evidence in favor of a direct link between the two measures, i.e., recovery of visual depth perception abilities assessed at the behavioral level, and normalization of binocular matching of orientation preference, as strongly suggested by the correlation between the two measures. Thus, the current study suggests that rescue of binocular matching might be a critical mechanism underlying the beneficial effects of vPL on visual depth perception.

At the visual cortical circuit level, other active training procedure, like voluntary physical exercise, are known to have an impact on GABAergic neural connections, with a selective and differential regulation in the activity of distinct sub-populations of interneurons (Fu et al., 2014; Sansevero et al., 2020). Specifically, amblyopia has been linked to increased numbers of active somatostatin positive interneurons, without any detectable effect on either vasointestinal protein or parvalbumin positive cells. In contrast, physical exercise was associated with both a specific increase of active VIP + cells, and a restoration to basal numbers of active SOM + interneurons, in agreement with the model put forward by Stryker and coll (Stryker, 2014). Even if a dissection of the possible changes at the GABAergic circuit level induced by vPL in amblyopic animals is beyond the scope of the present paper, future work will address this relevant issue.

In conclusion, the results reported in the present paper show that a non-invasive procedure of active training based on vPL under binocular sight conditions is very effective in favoring plasticity of the adult visual cortex and in eliciting recovery from amblyopia beyond the closure of the critical period. These results could have a direct impact on human health, opening new treatment possibilities for amblyopia and other still cureless neurodevelopmental disorders.

## Data Availability Statement

The raw data supporting the conclusions of this article will be made available by the authors, without undue reservation.

## Ethics Statement

The animal study was reviewed and approved by Health Ministry of Italy Aut. Numb. 144/2017-PR.

## Author Contributions

AS planned the experiments, coordinated the research, provided funding support, wrote the manuscript. AC, GS, CT, and ID performed the experiments and analyzed the data. NB provided funding support and contributed to manuscript writing. All authors contributed to the article and approved the submitted version.

## Conflict of Interest

The authors declare that the research was conducted in the absence of any commercial or financial relationships that could be construed as a potential conflict of interest.

## Publisher’s Note

All claims expressed in this article are solely those of the authors and do not necessarily represent those of their affiliated organizations, or those of the publisher, the editors and the reviewers. Any product that may be evaluated in this article, or claim that may be made by its manufacturer, is not guaranteed or endorsed by the publisher.
